# The Benefit of Pets and Animal-Assisted Therapy to the Health of Older Individuals

**DOI:** 10.1155/2014/623203

**Published:** 2014-11-16

**Authors:** E. Paul Cherniack, Ariella R. Cherniack

**Affiliations:** ^1^The Geriatrics Institute, University of Miami Miller School of Medicine, Division of Geriatrics and Gerontology, The Geriatrics and Extended Care Service and Geriatric Research Education, Clinical Center (GRECC) of the Miami Veterans Affairs Medical Center, Miami, FL, USA; ^2^Bruce W. Carter Miami VA Medical Center, Room 1D200, 1201 NW 16 Street, Miami, FL 33125, USA; ^3^Sha'arei Bina TAG, 1557 NE 164 Street, North Miami Beach, FL 33162, USA

## Abstract

Many studies utilizing dogs, cats, birds, fish, and robotic simulations of animals have tried to ascertain the health benefits of pet ownership or animal-assisted therapy in the elderly. Several small unblinded investigations outlined improvements in behavior in demented persons given treatment in the presence of animals. Studies piloting the use of animals in the treatment of depression and schizophrenia have yielded mixed results. Animals may provide intangible benefits to the mental health of older persons, such as relief social isolation and boredom, but these have not been formally studied. Several investigations of the effect of pets on physical health suggest animals can lower blood pressure, and dog walkers partake in more physical activity. Dog walking, in epidemiological studies and few preliminary trials, is associated with lower complication risk among patients with cardiovascular disease. Pets may also have harms: they may be expensive to care for, and their owners are more likely to fall. Theoretically, zoonotic infections and bites can occur, but how often this occurs in the context of pet ownership or animal-assisted therapy is unknown. Despite the poor methodological quality of pet research after decades of study, pet ownership and animal-assisted therapy are likely to continue due to positive subjective feelings many people have toward animals.

## 1. Introduction

Two-thirds of all US households [1, 2] and close to half of elderly individuals own pets [3]. Investigations involving pets and other animals attempting to improve the health of older individuals have involved many species, including dogs, cats, and manufactured simulations of animals [4]. In this paper, the evidence for the impact of animals on the health of the elderly is assessed. Given the small number of published manuscripts, a systematic review was not attempted. Rather, the studies considered were obtained by performing a PubMed search using terms including “pets, elderly, and animal-assisted.” Additional articles were obtained from the reference lists of the original articles found.

## 2. Potential Benefits of Animals

### 2.1. Effects on Mental Health

The most frequently studied use of animals with elderly participants has been to alleviate manifestations of cognitive disorders, such as agitation [5]. All of the studies were unblinded, not all were controlled, but most, though not all, showed small but statistically significant improvements in behavioral symptom scores in the animal-assisted interventions.

One trial, the sole study that used a bird, uniquely noted that animals conferred psychological benefits to cognitively unimpaired older individuals; 144 persons without cognitive impairment in nursing homes in Italy were exposed to either a canary, a plant, or neither of the two [6]. The individuals assigned to care for a canary or plant were provided with care instructions and participated in a three-month intervention, the details of which were not specified in the paper. Subjects who cared for the bird had significantly better scores at the end of the intervention on subscales of psychological symptoms in the Brief Symptom Inventory and LEIPAD-II-Short Version, which subjects in the other two groups did not.

Other investigations explored the effects of animals on demented elderly individuals (see Table 1). A dementia unit for US veterans piloted the use of a pet dog to elicit for socialization. Twelve demented patients exhibited a significant larger number of social behaviors, such as smiling or speaking in the presence of the dog, implying that animals might create benefit apart from any effect on cognition [7].

Another uncontrolled trial suggested that animals could help alleviate problematic behaviors in demented individuals. This trial enrolled elderly residents of two US nursing homes who had MMSE scores of 15 or below who were treated with animal-assisted therapy [8]. The participants, in a recreational room for one hour a day, met with a dog and its trainer. They could engage in a variety of activities including feeding, petting, grooming the animal, socializing with the trainer, and discussing pets the subjects previously owned. Subjects achieved a mean 25 percent, significantly better scores on the CMAI index of behavioral disturbance after the intervention.

Two further studies, in addition, piloted the efficacy of animal-assisted therapy on cognition and mood in cognitively impaired older persons. Twenty-five moderately demented residents of a nursing home were divided into two groups [9]. In the intervention group (mean Folstein Mini-Mental (MMSE) score 15.3, mean fifteen-question Geriatric Depression Scale (GDS) score 5.9), the subjects experienced a weekly hour and a half activity for 60 days in which they interacted with trained pet therapy dogs. The participants either walked, played with, petted, or held the animals under the supervision of a trainer. In the control group (mean MMSE score 18.3, mean GDS score 7.4, which was not significantly different than in the intervention group) the subjects watched the animals enter the nursing home but did not interact with them. Unfortunately, after the intervention, both groups increased their MMSE and lowered their GDS scores, but the changes in both groups between pre- and postintervention values were not significant. A second small study examined four moderately to severely demented residents of a nursing home who were videotaped for behavioral responses prior to and during an animal therapy session with a dog [10]. The residents displayed significantly fewer signs of agitation and more social behaviors during animal therapy.

An additional trial uniquely explored the possibility that animals might confer physical benefits to older persons with dementia and, furthermore, used fish, which did require the subjects to handle the animals. In this study, demented individuals in several nursing homes successfully gained weight after fish tanks were installed [11]. Sixty-two older persons who resided in the dementia units of three different nursing homes containing tanks in recreational and dining rooms that allowed a twenty 30 × 20 inch viewing area with background lighting to compensate for potential resident visual impairment were compared with another group of residents who had a “scenic ocean picture” added to similar rooms. Residents in each of the homes had different exposure times to either the fish tanks or the pictures. When the data from the subjects who were exposed to the fish tanks was pooled together, there was a mean 1.65 lb weight gain between three months before the tanks and four months after the tanks were placed (*P* < .000) but no gain in the control group.

Animals might provide other benefits to demented individuals, such as improving their ability to socialize, as suggested in several trials. In one study, which was not blinded, 33 individuals who lived in a nursing home were exposed to animals during 41.1 hours of animal-assisted therapy and 33.8 hours of recreational therapy without animals [12]. Long conversations between alert participants were more likely to occur in therapy groups when animals were present, but brief conversations were more likely when animals were absent. In another trial, a videotape captured the social interactions between 36 nursing home residents in ninety-minute occupational therapy sessions with or without a dog present [13]. Residents were more likely to have verbal interactions with the dog in the session. In a third investigation, thirteen demented residents were exposed to a plush mechanical toy dog that could sit up and wag its tail, or a robotic dog that could respond to seventy-five commands [14]. Subjects responded to both objects, similarly, by talking to it or clapping their hands when it moved.

Nurses have written their personal, qualitative observations that animals relieve loneliness and boredom, foster social interaction, and add variety to the lives of such persons, indirectly suggesting other possible advantages to human interactions with animals not thus far documented in clinical trials [5, 15]. In one survey, the nursing staff of an intermediate care unit delineated their perceptions of “cat mascots,” animals that spend the day in the unit [16]. There was no formal regulation of the interaction between the cats and the patients, nor any formal measures of the interaction. However, the nurses did state their opinions that the cats increased patient interactivity with their other people and their environment, and that the patients enjoyed their presence.

Pets may also positively influence the behavior of demented elderly owners. In one comparison survey, demented pet owners were less likely to exhibit verbal aggression but were otherwise similar to non-pet owners in likelihood of vegetative, hyperactive, or psychotic behaviors [17].

Thus far however, none of these studies on the use of animals in demented subjects have suggested a mechanism for how animals might alter the behavior of such individuals. One might speculate that animals might create a distraction to inhibit disruptive behavior or serve as a surrogate for human interaction to learn or practice social behavior.

Several investigations have also piloted the use of animals in the treatment of depression with mixed results. One small trial showed even a brief intervention conferred some benefit. Thirty-five individuals who were about to receive electroconvulsive therapy (ECT) spent 15 minutes with a dog and animal trainer or the same period of time reading magazines before ECT treatment sessions [18]. All subjects had both types of pretreatment every other day. Individuals reported lower levels of fear about the upcoming ECT rated on visual analogue scales when they had sessions with the dog. In a similar trial, forty-two depressed patients spent time waiting for ECT in rooms with or without aquariums. The presence of aquariums did not influence the pretreatment anxiety, fear, or depressive symptoms the patients experienced [19].

Animal-assisted therapy has been considered in the treatment of depression in institutionalized individuals in a number of studies. In one investigation, twenty-eight residents of an Italian nursing home had three-hour treatment sessions once a week for a month and a half with a cat or no change in their usual routine [20]. A nurse supervised individuals in a therapy room, who could pick up or play with the cat. The individuals who interacted with a cat did not have any significant difference in Geriatric Depression Screen score, or cognition as measured by MMSE, but did have sixteen-point lower systolic blood pressure (*P* = 0.05) and five-point lower diastolic blood pressure (*P* = 0.05) than subjects who were not exposed to the cat. In an additional survey, subjective rankings of pet attachment were actually associated with higher ratings of depressive symptoms in older individuals living in rural areas [21]. In another trial of 68 nursing home residents in Australia, individuals who visited a dog reported less fatigue, tension, confusion, and depression [22]. Cancer patients undergoing chemotherapy were divided into two groups, one of which had a weekly hour-long session of therapy with a dog and one of which did not [23]. Those patients at sessions at which a dog was present rated their symptoms of depression and anxiety half as severe as those who did not. Taken together, these studies imply a rather modest benefit at best for animals in depressed individuals.

A meta-analysis was conducted of five studies of the use of animal-assisted activities therapy in the treatment of depression in institutionalized subjects [24]. None of the five studies whose data was pooled for the meta-analysis was ever published in a scientific journal; four were printed in doctoral dissertations and the fifth was published in a book chapter almost thirty years ago. The meta-analysis concluded that such therapy could alleviate depressive symptoms with a “medium effect size.” Neither the meta-analysis nor the previously referenced manuscripts commented on possible mechanisms of an effect.

Other studies have examined if pets might assist the treatment of individuals with schizophrenia. Two investigations suggested that animals could improve social behaviors in elderly schizophrenics. Twenty schizophrenics, at least sixty-five years old, had three-hour visit every week for a year with a dog or cat and a therapist [25]. The subjects were taught to ambulate with the animals on a leash, bathe, feed, or groom them. A control group had a weekly news discussion session simultaneously with the animal therapy group. Schizophrenics exposed to animals had significantly improved mean scores on social functioning as part of the Social-Adaptive Functioning Evaluation scale which members of the control group did not. There were no differences between groups on survey instruments describing the subjects' impulse control or self-care.

In another investigation, 21 schizophrenic inpatients were divided into an intervention and control group [26]. Both had 45-minute meetings twice weekly with a psychologist for a total of 25 sessions. In the intervention group, a therapy dog and handler participated. The dog was the focus of interventions tailored to improved communication, social skills, and cognitive rehabilitation. The control group had similar sessions, except without the dog. Subjects in the intervention group had significantly better scores on the social contact score in of the Living Skills Profile and total score on the Positive and Negative Symptoms Score scale.

Not all investigations noted that schizophrenics derive benefit from animals. Fifty-eight older psychiatric inpatients in one trial were randomized to spend five sessions of either an hour a day with either pet therapy or an exercise group [27]. There was no difference in a forty-question psychiatric symptom score between groups. In addition to the trials of animal therapy in older persons with mental illness, qualitative research comprising focus groups of individuals recovering from acute episodes of psychiatric disease has outlined what subjects perceive to be benefits of pet ownership, such as companionship and a reinforced sense of self-worth [28]. However, subjects sometimes were troubled by their pet care responsibilities and grieved over the loss of pets.

Furthermore, several studies have implied that animals offer psychological or social benefits to the elderly independent of disease state. In one investigation, the effects of animals on the degree of loneliness of long-term care residents were assessed using a survey instrument [29]. Thirty-five people who lived in a nursing home had an experience in which, for two and a half months, they interacted with several animals including dogs, cats, and rabbits for two hours each [30]. They scored significantly higher on the Patient Social Behavior Score during and after the intervention. In another study, forty-five residents of three facilities were divided into those who received thirty-minute animal-assisted therapy once a week for a month and a half, the same therapy three times a week, or not at all. Residents who received any animal therapy scored significantly lower on the UCLA Loneliness Scale than those who did not. In a case series, a robotic dog improved the loneliness scores on one assessment instrument of five medically ill elderly persons [31]. In a qualitative survey, dog owners over age of 70 in Austria stated that dogs provided companionship and a sense of purpose [32]. However, finally, in few cases, animal-assisted therapy has even been utilized to provide subjective benefit to critically ill patients in intensive care units [33].

### 2.2. Effects on Physical Health

Numerous studies have recorded evidence of the effects of animals on the physical health of elderly individuals. Several have attempted to quantify physiological benefits of the presence of animals on the effects of stress (see Table 2). One study exposed hypertensive pet owners to the stress of solving an arithmetic problem and making a speech [34]. The investigators instructed half of the subjects to acquire a pet, and the total subject population was restudied after six months. Those who owned a pet had significantly lower increases in systolic and diastolic blood pressure in response to the stressor than those who did not. In an additional investigation, the presence of a dog in the room alleviated an increase in blood pressure in response to the stress of public speech [35]. Eleven community-dwelling older individuals with hypertension, mean age 81.3, were asked to speak in the presence or absence of a dog while blood pressure was being recorded. Participants who spoke in the presence of a dog had a significantly lower diastolic blood pressure (mean difference = 12.8 mmHg, *P* = 0.006) than in the absence of the dog. Another 10 healthy dog owners of a canine achieved a significant systolic and diastolic blood pressure reduction and subjective measures of anxiety after performing a stressful task whether their own dog or not was used [36]. There was a greater improvement of outcome measures when the subject's own dog was used, which lasted up to an hour. Finally, in a small case series of community-dwelling elderly individuals aged 65 to 91, one group of participants received a weekly visit from a nurse with a dog for a month, while one group had visits without the dog [37]. Those who were in contact with the dog had a significantly lower mean systolic and diastolic blood pressure than those who did not (mean decrease 8 mmHg systolic and 4 mmHg diastolic, *P* < .01 difference in the intervention group from baseline). Taken together, these investigations imply ameliorating effect of pet ownership on the physiologic effects of stress.

Epidemiologic studies suggest pet owners may acquire physical benefits, such as improved blood pressure and greater physical activity. Among 5741 individuals in Australia, those who possessed pets had a significantly lower resting systolic blood pressure, a mean 5 mg/dL lesser cholesterol, and 84 mg/dL triglyceride levels which were statistically significant [38]. In another survey of 1179 elderly persons (mean age 70), pet owners had comparatively reduced systolic mean arteriolar and pulse pressure, and lesser risk of hypertension (O.R. = 0.62) [39].

Other investigations imply that dog walking encourages individuals to take part in physical activity (see Table 3). In another study, dog owners in Canada (not exclusively elderly, but including participants up to age 80) were more likely to visit multiuse or walk-through parks than individuals who did not possess dogs [40]. An investigation of 5902 individuals in the US noted a positive relationship between dog walking and amount of total walking time [41]. Dog owners were more likely to walk at least 150 minutes a week (O.R. 1.69; 95% CI 1.13–1.59) and were more likely to involve themselves in any physical activity during leisure time (O.R. 1.69; 95% CI 1.33–2.15). Dog walking was also associated with likelihood of walking in 608 Washington state residents (*P* < .01) [42]. A recent analysis of a cohort of 545 Scottish participants, all at least 65 years old, dog owners were more likely to report themselves at the highest level of physical activity than those not possessing dogs [43]. Among 3,075 elderly individuals (aged 70–82) in Memphis and Pittsburgh, dog owners were twice as likely but non-dog owners half as likely to take part in physical activity compared to people who did not own pets [44].

Dog walking may encourage participants to take part in other beneficial physical activities and to preserve their functionality. In the largest survey to date, the California Health Interview Survey, comprising more than 55,000 individuals, dog owners more commonly walked as a leisure time activity than those who did not own a pet (O.R. 1.6; 95% CI 1.5–1.8) but were less likely to walk for transportation (O.R. 0.91; 95% CI .85–.99) [45]. In an epidemiological survey of more than one thousand elderly persons at least 65 years old in Canada, the loss of ability to perform activities of daily living of persons who did not own pets progressed at a greater rate than for pet owners [46]. In a Japanese survey of 5283 adults up to age of 79, dog owners were 1.54 times more likely to obtained recommended amounts of physical activity [47]. Among 127 elderly persons in Colorado, those possessing pets ambulated longer distances (*P* < .05) and had lower triglycerides (*P* < .01) than those without animals [48].

However, dog ownership may not be enough to guarantee greater physical activity. In one Australian study, owners of large dogs spent more time walking than those who owned small dogs, and dog ownership* per se* was not associated with greater probability of obtained recommended activity levels [49]. While none of the manuscripts considering the effect of dog walking on physical activity specifically considered mechanism, one might speculate that, rationally, the need to walk a dog might create a need to walk more, and that increased physical activity might be more associated with the pet's needs than those of their owners.

Pet ownership may confer additional benefits to patients with cardiovascular disease (see Table 4). Participants in a treatment trial of antiarrhythmia drugs who owned dogs were less likely to die over a year than others, including those who owned other types of animals [50]. Patients owning pets who were released from a coronary care unit were significantly more likely to survive after one year [51]. Individuals who had sustained a myocardial infarction in the past year and walked their dogs for fifteen minutes three times daily improved their exercise capacity on stationary bicycles (*P* < .05) [52]. Further analysis of a trial in which 460 pet owners were implanted with a defibrillator (mean age = 61) revealed that possession of pets rendered participants less likely to die (*P* = 0.036) in the following 2.8 years [53]. In another survey, seventy-six persons with congestive heart failure were divided into three groups, one of whom visited a dog for 12 minutes, one of whom visited a person for 12 minutes, and one of whom did not receive either [54]. Those who were exposed to the dog had a lower systolic pulmonary artery or capillary wedge pressures, and reduced serum epinephrine concentrations. Sixty-nine in-patients with congestive heart failure participated in an ambulation training program in which they walked with a dog and a trainer [55]. When matched with a “historical sample” of congestive heart failure patients, subjects who walked with a dog walked twice as far as the “historical sample” (mean 230.07 steps/day versus 120.2 steps/day, *P* < .0001). Not all studies imply that pets are beneficial for cardiovascular disease; in one follow-up study of patients admitted to a unit for “acute coronary syndrome” those owning a pet were more prone to death or rehospitalizations a year later [56]. Nevertheless, given the preponderance of the evidence, the American Heart Association has released a statement acknowledging the relationship and causality of pet ownership in the attenuation of cardiovascular disease risk [57].

## 3. Harms of Animals

While the use of pets and animal therapy might confer several potential health benefits to older persons, harms also exist. Pet owners fall and sustain fractures as a result of their animals. The US Center for Disease Control and Prevention noted that there were 86,629 falls a year attributed to dogs and cats, with a mean injury rate of 29.7 per 100.000 persons a year from 2001 to 2006 [58]. Older persons above 75 had the highest injury rates (68.8 for those 65–74, and 70.6 for those 75 and older), twice as high as those between 35 and 44 (28.6). A case series from Australia also reported 16 fractures to elderly individuals who were at least aged 65 [59]. Most of the injured were women, and individuals commonly tripped over the pets or fell while bending down to feed them. The pets were most commonly dogs and cats, but they also included birds, a goat, and a donkey.

Other harms may be present, as well. Pets can be expensive, time-consuming, and complex to care for. The average lifetime cost of an average-sized dog can be $10,000 and a cat $8,000 [1]. The pets need adequate food, housing, hygiene, and veterinary care [60]. Elderly persons may, because of physical or cognitive limitation, be less able to provide such care than younger persons. In addition, the pets might damage an elderly person's property, although there are no reports in the published medical literature. Pets that are not safeguarded properly by their owners might also be a threat to other people and to the environment. The pets could potentially injure others, harm their property, or create fear or mistrust. The animals might damage the environment (e.g., destroying animals and plants, creating waste).

Institutionalized elderly may also be less able to interact appropriately with animals. One qualitative report of the reactions of staff to an institutional cat mascot stated that residents placed the cat in garbage and toilet and nearly ran over its tail with wheelchairs [16].

Animals have the potential to cause human infection and trauma. Concern about human infections caused by pets has been mentioned as a possible adverse consequence to pet ownership in the elderly [61]. Greater than 200 different zoonotic infections exist [62]; however their exact incidence in the elderly who own pets or participate in animal-assisted treatments has not been documented and remains unknown. Similarly, there may be traumatic injury from animal bites or scratches, but similarly, how frequently this takes place as well as the impact of any events is uncertain. The aforementioned report of an institutional cat mascot mentioned that a cat scratched a patient but did not give further details as to this or other human injuries [16].

Pets might also cause psychological harm. Humans can become very attached to their pets, and when they lose them, they may undergo grief reactions similar to those with loss of other people [32, 60]. The results of any investigations of such losses on human health in the elderly have not been published.

## 4. Future Directions and Conclusions

Preliminary studies have suggested the potential benefits of animals on the physical and psychological health in humans. Despite over four decades of research, these studies remain preliminary. They are compounded by methodologic problems including small sample size and lack of adequate controls and blinding. A review of animal research more than a decade ago outlined barriers [63] that still need to be overcome, including access of animals to subjects in institutional settings, fear of zoonotic diseases, lack of standardized survey instruments, and recruitment of animal handlers. There have yet to be blinded animal investigations.

In addition, the potential influence of the differences in demographic characteristics of human subjects (e.g., differences in education, ethnicity, and income) remains uncertain. In one study, elderly Latino pet owners, mean age 66, responded to a survey of their attitudes toward their dogs and health [64]. Two-thirds considered the dogs to be their “best friends” and “reason for getting up in the morning” and their health to be better “than most people,” and seventy-five percent deemed their health “excellent.” Future investigations can clarify such influences.

Thus far, studies on the effects of animals on both mental and physical health have reported modest benefits. Trials of animal-assisted therapy demonstrated improvements in behavioral symptom scores in small numbers of subjects of limited duration. Investigations on the influence of animals on physical health, particularly epidemiological studies, that imply that the presence of animals can reduce cardiovascular risk, are more robust methodologically, but prospective trials demonstrating clinical benefit still need to be performed. New uses of animals may be piloted in the future. For example, in one preliminary report, a dog was trained to detect human melanomas by smell [65]. The use of animals as pets and in therapy may also have harms, but their incidence is rare, and these hazards have been even less well documented than the benefits. There has been no formal determination if whether these benefits outweigh the costs of feeding and caring, which are listed for comparison in Table 5. However, many reports describe participants' subjective positive feelings towards animals. These positive subjective feelings that people have toward animals together with growing evidence of a potential role in the treatment of cardiovascular disease may motivate their continued use of therapy and ownership.

## Figures and Tables

**Table 1:** Studies on use of animals in dementia.

Study	Type of study	*N*	Summary of results
Kongable et al. [7]	Case series/observational	12	Demented subjects had more social behaviors in presence of animal

Richeson [8]	Case series/observational	15	Animal therapy reduced amount of behavioral disturbance

Moretti et al. [9]	Controlled, unblinded, prospective	21	No difference between control and intervention, both had improved MMSE and lower GDS scores

Sellers [10]	Case series/observational	4	Subjects had less agitation and more social behavior with pet present

Edwards and Beck [11]	Case-control/prospective	62	Subjects exposed to a fish tank had greater weight gain (*P* < .000)

Bernstein et al. [12]	Self-controlled, prospective, observational	33	Longer conversations in subjects with animals present

Fick [13]	Self-controlled, prospective, observational	36	More social behavior when animals present

Tamura et al. [14]	Controlled, unblinded, prospective, observational	13	Social response similar to real or toy dog

**Table 2:** Studies on the use of animals on blood pressure.

Study	Type of study	*N*	Summary of results
Allen et al. [34]	Randomized, unblinded, prospective, controlled	48	Subjects had lower blood pressure and heart rates in response to an acute mental stressor in the presence of a pet

Friedmann et al. [35]	Controlled, unblinded, prospective	11	Blood pressure lower when animal and acute mental stressor present

Barker et al. [36]	Controlled, unblinded, prospective	10	Pet owners reduced systolic and diastolic blood pressure in the presence of animal when mental stressor present

Anderson et al. [38]	Retrospective chart survey	5741	Pet owners had lower resting blood pressure

Wright et al. [39]	Mail questionnaire	1179	Pet owners had lower systolic, mean arteriolar, pulse pressures, risk of HTN (O.R. = 0.62)

**Table 3:** Studies on the use of animals on physical activity.

Study	Type of study	*N*	Summary of results
Temple et al. [40]	Prospective, observational	48	Pet owners more likely to use parks

Reeves et al. [41]	Telephone survey	5902	Dog walkers most likely to perform leisure-time physical activity

Moudon et al. [42]	Telephone survey	608	Dog owners more likely to walk (O.R. = 1.69)

Thorpe et al. [44]	In-person questionnaire survey	3075	Dog owners more likely to engage in physical activity, walking

Feng et al. [43]	Prospective, observational	545	Dog walking associated with physical activity (measured by accelerometer carried by subjects)

Yabroff et al. [45]	Telephone survey	41,514	Dog owners walked longer times

Raina et al. [46]	Telephone survey	1054	Pet owners experienced slower deterioration in activities of daily living

Oka and Shibata [47]	Online survey	5253	Dog walkers had more physical activity

Dembicki and Anderson [48]	Cross-sectional, observational study	127	Dog owners walked longer times

Schofield et al. [49]	Telephone survey	1237	Dog ownership not associated with recommended physical activity large dog owners walked more than small dog owners

**Table 4:** Studies on the use of animals in cardiovascular disease.

Study	Type of study	*N*	Summary of results
Friedmann and Thomas [50]	Retrospective data analysis	424	Dog owners less likely to die 1 year after MI

Friedmann et al. [51]	In-person interview survey	96	Pet owners had higher 1-year survival after CCU discharge

Ruzic et al. [52]	Prospective, controlled, unblinded, longitudinal study	59	Subjects walking dogs regularly achieved a higher workload on a bicycle exercise test (*P* < .06)

Friedmann et al. [53]	Retrospective data analysis	460	Pet owners implant with defibrillator more likely to survive

Cole et al. [54]	Randomized, controlled, unblinded study	76	Subjects exposed to an animal had significantly better hemodynamic and neurohormonal parameters

Abate et al. [55]	Observational intervention group, historically case-controlled study	69	Subjects with dog-assisted ambulation walked significantly greater distance

Parker et al. [56]	In-person interview survey	424	Pet owners more likely to have cardiac morbidity and mortality one year after admission for an acute coronary syndrome

**Table 5:** Potential benefits and risks of animals in the elderly.

Potential benefits	Potential harms
Increased physical activity	Cost
Improved survival in cardiovascular disease	Injury to self
Improved circulatory hemodynamic responses	Injury to others
Less behavioral disturbance in demented patients	Damage to property
Improved socialization in demented patients	Damage to environment
Weight maintenance in demented patients	Zoonotic infections
Less anxiety, fear in depressed patients	Adverse psychological event (e.g., grief reaction over loss of pet)
Improved social behavior in schizophrenics	Adverse social event (e.g., friends, neighbors fear pet)
Less loneliness	Greater rehospitalization rate in acute coronary syndrome patients
